# Soluble B and T Lymphocyte Attenuator Correlates to Disease Severity in Sepsis and High Levels Are Associated with an Increased Risk of Mortality

**DOI:** 10.1371/journal.pone.0169176

**Published:** 2017-01-05

**Authors:** Anna Lange, Jonas Sundén-Cullberg, Anders Magnuson, Olof Hultgren

**Affiliations:** 1 Department of Infectious Diseases, School of Medical Sciences, Örebro University, Örebro, Sweden; 2 Division of Infectious Diseases and Center for Infectious Medicine, Karolinska Institute at Karolinska University Hospital Huddinge, Stockholm, Sweden; 3 Clinical Epidemiology and Biostatistics, School of Medical Sciences, Örebro University, Örebro, Sweden; 4 Department of Microbiology and Immunology, School of Medical Sciences, Örebro University, Örebro, Sweden; King Abdullah International Medical Research Center, SAUDI ARABIA

## Abstract

**Introduction and aims:**

B- and T-lymphocyte Attenuator (BTLA), Cytotoxic T-lymphocyte-associated protein 4 (CTLA-4) and Programmed Death 1 (PD-1) are co-inhibitory receptors that regulate T cell activation. In the present study of ICU-treated patients we measured plasma concentrations of their soluble isoforms, with the aim to evaluate their potential as sepsis biomarkers and utility as prognostic indicators.

**Methods:**

101 patients with sepsis, 28 patients with non-infectious critical illness (ICU controls) and 31 blood donors (healthy controls, HC) were included in the study. Plasma concentrations of soluble BTLA (sBTLA), CTLA-4 (sCTLA-4) and PD-1 (sPD-1) were measured with ELISA in serial blood samples. Comparisons were made with Mann-Whitney *U* test and correlations were assessed with Spearman’s Rank correlation test. Cox proportional hazard models, with sBTLA and sPD-1 as fixed and sBTLA as time-varying covariates, were used to determine association with 28-day mortality.

**Results:**

sBTLA levels were significantly higher in the sepsis cohort (median 14 ng/mL, IQR 8–29) compared to ICU controls (9 ng/mL, IQR 5–26, p = 0.048) and HC (2.9 ng/mL, IQR 0.9–9.1, p<0.01), and correlated to SOFA score. sBTLA levels were higher in 28 day sepsis non-survivors than in survivors (baseline median 28 ng/mL, IQR 13–41 vs 13 ng/mL, IQR 8–23, p = 0.04). After adjustment for age and comorbidities, the relative risk of 28 day mortality was nearly 5-fold higher in sepsis patients with a baseline sBTLA > 21 ng/mL, compared to those with a level below this threshold. sBTLA was even more associated with mortality in the time-varying analysis. sPD-1 levels were lower in the sepsis cohort compared to HC but not compared to ICU controls and were not associated with mortality. sCTLA-4 was detectable in only one subject.

**Conclusion:**

Plasma concentrations of soluble BTLA were increased early in sepsis/septic shock and correlated to severity of disease. A baseline concentration >21ng/mL was associated with a poor prognosis.

## Introduction

Despite decreasing mortality rates over the past two decades, severe sepsis and septic shock still belong to the most serious acute medical conditions and are leading causes of death in the ICU [1, 2]. While most patients who make it through the acute phase recover quickly, some enter a prolonged state of organ dysfunction, run a risk of super-infections and often fail to clear infections, a condition referred to as immunoparalysis [3–6]. Adding further complexity to this process, although pro-inflammation often dominates in the early sepsis phase, there is simultaneous anti-inflammatory cytokine activity, and T cell activation and proliferation occurs in parallel with increased apoptosis [7–9].

Antigen-specific activation of naïve T lymphocytes requires double signals; T cell receptor (TCR) recognition of antigen bound to MHC on Antigen presenting cells (APCs), and a second signal delivered through the interaction between the co-receptor CD28 and its cognate ligands CD80 and CD86 on the same APC [10]. The magnitude of the immune response is regulated by an intricate network of co-signaling cell-surface bound receptors and their corresponding ligands. Programmed Death 1 (PD-1), Cytotoxic T Lymphocyte-associated Antigen 4 (CTLA-4) and B- and T-lymphocyte Attenuator (BTLA) are three well characterized co-inhibitory receptors, through which cell activation and proliferation can be abated. CTLA-4 competes with CD28 for interaction with the CD80 and CD86 ligands on APCs, and by interfering with co-stimulation CTLA-4 can have an attenuating effect on T cell activation [11]. PD-1 is expressed on T-cells upon activation, and has two ligands; PD-L2, which is limited to APCs, and PD-L1, which is expressed on various immune cells [12]. BTLA is broadly expressed: on B and T lymphocytes, macrophages, dendritic cells and NK cells and can be either up- or down-regulated after stimulation depending on the cell type [13]. Its ligand Herpes Virus Entry Mediator (HVEM) is also expressed on both sides of the APC-lymphocyte entity. BTLA-HVEM as well as PD-1-PD-L1/PD-L2 interactions may convey an inhibiting signal [12, 14]. Negative co-stimulatory pathways are important both in the defense against infection and in maintaining peripheral tolerance and immune homeostasis, which is illustrated by the fact that mice deficient in PD-1, BTLA or CTLA-4 develop autoimmune and lymphoproliferative disease [15–17]. While increased PD-1 expression on T cells is an established marker of immunoparalysis in sepsis, the roles of CTLA-4 and BTLA remain to be determined [18–20]. BTLA and PD-1 expression was shown to be altered on peripheral blood T cells from septic patients. Increased PD-1 expression has also been suggested as a marker of increased risk for nosocomial infections and of a poor prognosis [18, 21]. Experimental models have demonstrated that blockade of the PD-1 and CTLA-4 pathways can improve outcome in sepsis [22–25].

It has been established that there are soluble isoforms of the co-inhibitory receptors, lacking the transmembrane region, and it was shown in vitro that they can antagonize negative co-signaling by competitively binding the corresponding ligand of their membrane-bound equivalents [26–28]. While a recombinant CTLA fusion protein (abatacept), that interferes with the co-stimulatory signal required for T cell activation, has been used for treatment of rheumatoid arthritis unresponsive to anti TNF treatment since 2005, the biological activity of soluble co-inhibitory receptors still remains to be discovered. To examine their participation in immune regulation, we aimed to measure plasma concentrations of the soluble isoforms of PD-1, CTLA-4 and BTLA in critically ill patients and evaluate their usefulness as sepsis biomarkers and indicators of severity of disease and prognosis.

## Methods

### Setting and study population

Inclusion criteria were: a diagnosis of severe sepsis or septic shock within the last 24 hours in patients admitted to, or already in, the mixed intensive care unit of Karolinska University Hospital in Huddinge. Severe sepsis and septic shock were defined according to the criteria proposed by the American College of Chest Physicians/Society of Critical Care Medicine [29]. Sepsis was defined as an infection in the presence of a systemic inflammatory reaction (SIRS). SIRS was defined by at least two of the following criteria: 1) body temperature higher than 38°C or lower than 36°C, 2) heart rate higher than 90/min, 3) respiratory rate higher than 20/min or PaCO2 lower than 32 mmHg, 4) white blood cell count higher than 12000 or lower than 4000 cells/μl. Severe sepsis was defined as sepsis associated with organ dysfunction, hypoperfusion or hypotension and septic shock was defined as sepsis with persistent hypotension despite adequate fluid resuscitation.

Control patients were defined as patients admitted to the ICU for non-infectious critical illness within the last 24 hours, and with an expected length of stay in the ICU longer than 24 hours. Exclusion criteria in both cohorts were age <18 years.

A total of 130 patients were enrolled in the study between Oct 2005 and June 2009. After review of complete patient charts and laboratory and microbiological tests, one control patient was reclassified as septic shock and one as severe sepsis; two septic shock patients were reclassified as severe sepsis, and one as control. Finally, one patient was excluded from analysis altogether because a clear classification proved impossible. This left 101 patients with severe sepsis (n = 14) or septic shock (n = 87) (sepsis cohort) and 28 patients who were ICU-treated for other critical conditions (ICU-controls). Patients were also reclassified according to The Third International Consensus Definitions for Sepsis and Septic Shock (Sepsis-3) [30]. According to this, sepsis is defined as life-threatening organ dysfunction (defined as an increase of 2 points or more in the Sequential Organ Failure Assessment (SOFA) score) due to a dysregulated host response to infection. Septic shock is defined as sepsis and persistent hypotension (despite adequate volume resuscitation) requiring vasopressors to maintain MAP greater than or equal to 65 mm Hg and a lactate greater than or equal to 2 mmol/L. With Sepsis-3 definitions 23 patients within the sepsis cohort had sepsis and 78 had septic shock. 31 blood donors enrolled between 2010 and 2013 served as healthy control subjects (healthy controls, HC).

### Patient characteristics

Clinical data was recorded for study patients, and age and sex for healthy control subjects. Acute Physiology and Chronic health Evaluation (APACHE) score was determined on admittance to the ICU and severity of disease was assessed daily with Sequential Organ Failure Assessment (SOFA) score. Blood and other relevant cultures were performed and the results of these, as well as of biological parameters, were recorded.

### Ethics

The study was conducted in accordance with the declaration of Helsinki. It was approved by the regional ethical review board of Stockholm, ref. 217/03. A written informed consent was obtained from study subjects or next of kin.

### Plasma samples

Blood samples for routine biochemical tests and plasma for later analyses were collected from study patients at inclusion and at 24, 48, 96 and 144 hours and kept at -80°C until used. Healthy controls were sampled in connection with blood donation and plasma was stored correspondingly.

### ELISA analyses

sPD-1 was analyzed at 1:2 dilution with RayBio Human PD-1 ELISA Kit, detection range 24.58–6000 pg/mL, RayBiotech, Inc. sBTLA was analyzed at 1:5 dilution with Human BTLA ELISA Kit, detection range 0.47–30 ng/mL, Cusabio. Plasma sCTLA-4 was analyzed at 1:2 dilution with Human sCTLA-4 Platinum ELISA, detection range 0.16–10 ng/mL, eBioscience. All ELISA analyses were performed according to the manufacturers’ instructions.

### Statistical analysis

**Fig 1:**
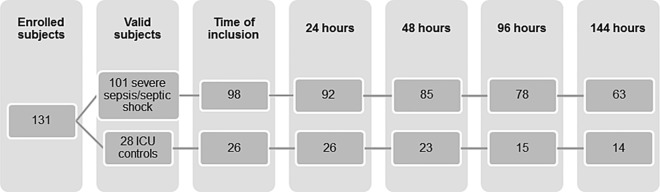
Plasma samples available for analysis over the course of the study. Day of inclusion: Sepsis cohort; 3 missed samples. ICU controls; 2 missed samples. At 24 hours: Sepsis cohort; 4 dead, 4 transferred to other ICU:s and 1 discontinued the study. ICU controls; 1 dead, 1 transferred to another ICU. At 48 hours: Sepsis cohort; 3 transferred to other ICU:s and 4 missed samples. ICU controls; 1 dead, 1 transferred to another ICU and 1 missed sample. At 96 hours: Sepsis cohort; 4 dead, 2 transferred to other ICUs, 2 discontinued and 4 missed samples. ICU controls; 5 dead patients and 5 missed samples. 144 hours: Sepsis cohort; 1 dead, 1 transferred to another ICU, 3 discontinued the study, 4 were discharged from the ICU and 8 missed samples. ICU control; 2 dead patients and 3 missed samples.

IBM SPSS Statistics for Windows, Version 22.0. Armonk NY: IBM Corp and Stata Statistical Software: Release 14. College Station, TX: StataCorp LP were used for statistical calculations. Plasma concentrations were summarized with median and interquartile range (IQR), according to data distribution. Comparison between the three study cohorts were made by Kruskal-Wallis test followed by pairwise group Mann-Whitney test corrected for multiple testing in the strong sense with the Bonferroni-Holm method [31]. Subgroups were compared with Mann-Whitney *U* test. Linear regression with baseline markers on log10 scale as outcome was used to evaluate age-adjusted differences between study groups. Correlations were analyzed with Spearman’s rank correlation test and presented as Spearman’s rho. Logistic regression was used to estimate the ability of biomarkers to diagnose sepsis and to predict mortality, by calculating the area under the Receiver Operating Characteristic (ROC) curve (AUC) of the sensitivity and 1-specificity. Risk of mortality was presented with the Kaplan-Meier method. Subjects were followed from inclusion until day 28 or the day of death, with no censoring. 3 patients were not sampled on the day of inclusion and were not included in the survival analysis. Cox proportional hazard models were used to evaluate if baseline levels of biomarkers were associated with mortality, adjusted for a) age, b) age and baseline comorbidities that were judged to be possible confounders (cardiovascular disease, chronic heart failure, COPD, diabetes, renal failure, liver disease and malignancy) on a categorical scale: 0, 1 and ≥2 comorbidities, due to no events (death) in patients with COPD or liver disease or more than 2 comorbidities, and 3) age, comorbidities and s-lactate. Biomarkers were evaluated on continuous log10 scales, as well as on categorical scales which were created as follows: The baseline concentration of the respective marker in the sepsis cohort was divided into three categories based on the 33^rd^ and 67^th^ percentile. To assess the association between sBTLA as a dynamic marker and mortality, we used a time-varying Cox model with sBTLA as the time-varying risk factor. Due to death, transfer to other ICUs and to medical or surgical wards because of recovery, as well as loss because of hemolysis and missed sampling, the number of plasma samples available for analysis diminished over the study period as demonstrated in (Fig 1).

Missing sBTLA data were imputed with multiple imputation (MI) technique implemented in the software Stata, based on the concepts of Rubin [32]. Day of sample, age, sex and baseline data; comorbidities (hypertension, cardiovascular disease, chronic heart failure, COPD, diabetes, renal failure, liver disease and malignancy), immunocompromised or not, acquisition of infection (community or hospital), SOFA-score and APACHE II score were used as predictors for the log sBTLA as well as for the categorical sBTLA imputation model. Association measures were hazard ratios (HR) with 95% confidence intervals (CI). P-values less than 0.05 were considered statistically significant.

## Results

### Characteristics of the study population

In the sepsis cohort 51.5% had a community-acquired and 48.5% a hospital-acquired infection, defined as onset of infection later than 48 hours after hospitalization, within 72 hours after hospital discharge or within 30 days after surgery. As defined by APACHE II criteria: had received immune-suppressive therapy, chemotherapy, radiation, long term or recent high dose steroids or had a disease sufficiently advanced to suppress resistance to infection, 28% in the sepsis cohort and 4% (one patient) among ICU controls were immunocompromised at baseline [33]. The types of immunosuppression in the sepsis cohort were; chemotherapy (n = 14), immunosuppressive therapy due to solid organ transplant (n = 8), autologous/allogeneic stem cell transplantation (n = 2), immunosuppressive therapy due to other disease (n = 2), neutropenia (n = 1) and HIV infection (n = 1). 44% had recent acute or elective surgery. The most common focus of infection was abdominal (41%) followed by pulmonary (24%) and urinary tract (15%). A positive blood culture was found in 48% of sepsis patients. Among ICU controls the most common diagnosis was cardiac arrest. In this cohort, 43% developed an infection during the study period (median 2.5 days) (Table 1).

**Table 1:** Characteristics of the study population.

	Sepsis	ICU-controls	Sepsis vs ICU controls p-value	Healthy controls
# of patients	101	28		31
Severe sepsis/septic shock	14/87			
Sepsis-3 def., sepsis/septic shock	23/78			
Age	63 (14.5)	60 (9.9)	*	50 (7.5)
Sex male/female, n (male %)	55/46 (55)	19/9 (68)		24/7 (77)
APACHE II score	21 (8.7)	22 (7.5)	p = 0.7	
SOFA score on inclusion	10 (3.4)	9 (3.9)	p = 0.4	
**Biological parameters**				
Leukocyte count (x10^9^ Cells/L)	14.9 (11.0)	11.3 (9.7)		
CRP (mg/L)	231 (111)	47 (64)		
S-Lactate (mmol/L)	4.3 (3.8)	3.5 (2.7)		
**Chronic health status**				
Hypertension	24 (24)	8 (29)	p = 0.6	
Cardiovascular disease	10 (10)	6 (21)	p = 0.1	
Congestive heart failure	6 (6)	1 (4)	p = 1.0	
Diabetes	16 (16)	9 (32)	p = 0.05	
COPD	4 (4)	2 (7)	p = 0.6	
Renal failure	3 (3)		p = 1.0	
Liver disease	7 (7)	5 (18)	p = 0.1	
Malignancy	36 (36)	5 (18)	p = 0.07	
Immuno-compromised	28 (28)	1 (4)	p<0.01	
**ICU control category**				
Cardiac arrest		11 (39)		
Liver tx or other major surgery		5 (18)		
Bleeding esophageal varices		3 (11)		
Myacardial infarction		3 (11)		
Other hypovolaemic shock		1 (4)		
Intoxication		1 (4)		
Diabetic ketoacidosis		1 (4)		
Massive pulmonary embolism		1 (4)		
Liver encephalopathy		1 (4)		
Respiratory failure		1 (4)		
**Acquisition of infection**				
Community	52 (51.5)			
Hospital	49 (48.5)			
ICU		12 (43)		
**Focus of infection**				
Abdominal	41 (41)			
Pulmonary	24 (24)			
Urinary	15 (15)			
Skin/skeletal	9 (9)			
Neutropenia	2 (2)			
Pulmonary + meningitis	1 (1)			
Other/unknown	9 (9)			
**Positive blood culture**				
Total	48 (48)	4 (14)		
*E*. *coli*	16 (16)	1 (4)		
enterococci	6 (6)			
*Staphylococcus aureus*	5 (5)			
*Streptococcus pneumoniae*	3 (3)			
*Klebsiella*	3 (3)			
*Pseudomonas aeruginosa*	3 (3)			
*Serratia marcescens*	2 (2)			
*Neisseria meningitidis*	1 (1)			
*Candida albicans*	1(1)			
polymicrobial	1 (1)			
other gram positive species	4 (4)	3(3)		
other gram negative species	2 (2)			

Data is presented as mean (SD) and n (%) unless otherwise stated.

* Sepsis/ICU controls p = 0.19, sepsis/HC p<0.01, ICU controls/HC p<0.01 (adjusted for multiple comparisons).

### Comparison of sBTLA and sPD-1 in septic patients, ICU controls and healthy controls

**Fig 2:**
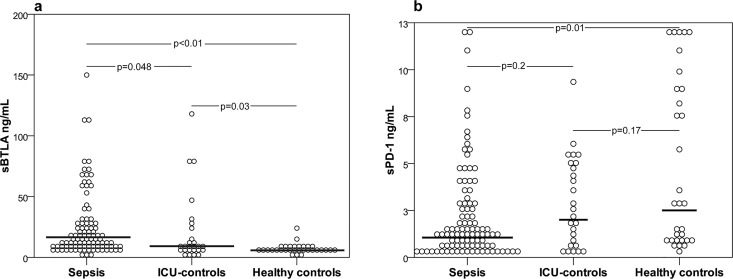
Plasma sBTLA and sPD-1 concentrations at baseline in the sepsis cohort, ICU controls and blood donors. (a) sBTLA concentrations in the sepsis cohort (median 14 ng/mL, IQR 8–29), ICU controls (median 9 ng/mL, IQR 5–26) and HC (median 5 ng/mL, IQR 5–8). (b) sPD-1 concentrations in the sepsis cohort (1.3 ng/mL, IQR 0.6–5.8), ICU controls (2.4 ng/mL, IQR 0.7–5) and HC (2.9 ng/mL, IQR 0.9–9.1). Bars indicate median concentrations. Linear regression with markers on log10 scale as outcome gave the following age-adjusted p-values: sBTLA: sepsis vs HC (p<0.01), ICU controls vs HC (p = 0.02) and sepsis vs ICU controls (p = 0.03). sPD-1: sepsis vs HC (p<0.01), ICU controls vs HC (n.s.) and ICU controls vs sepsis (p = 0.27). P-values in figure and text are adjusted with the Bonferroni-Holm method.

The Kruskal-Wallis test showed significance for differences between groups for the baseline concentration of sBTLA (p<0.01) and sPD-1 (p<0.01). sBTLA plasma concentrations were highest in the sepsis cohort (median 14 ng/mL, IQR 8–29), significantly lower in ICU controls (median 9 ng/mL, IQR 5–26, p = 0.048) and lowest in HC (median 5 ng/mL, IQR 5–8, compared to the sepsis cohort p<0.01, to ICU controls p = 0.03). Significant differences were still present after age adjustment (Fig 2A).

sPD-1 concentrations were significantly lower in the sepsis cohort compared to HC (1.3 ng/mL, IQR 0.6–5.8, vs 2.9 ng/mL, IQR 0.9–9.1, p = 0.01) but there was no significant difference compared to ICU controls (2.4 ng/mL, IQR 0.7–5, p = 0.2) or between ICU controls and HC (p = 0.17). Significant differences were still present after age adjustment (Fig 2B). In terms of predictive ability to diagnose sepsis in the ICU-treated cohort (sepsis + ICU controls), AUC for sBTLA and sPD-1 were 0.626 (95% CI 0.492–0.76, p = 0.048) and 0.603 (95% 0.477–0.730, n. s) respectively. sCTLA-4 was analyzed in plasma sample series from 16 patients in the sepsis cohort, 4 ICU-controls and 5 blood donors. We found a detectable, but low level of sCTLA-4 in only one sepsis plasma sample (0.8 ng/mL) and in no sample from the other two cohorts. Therefore we discontinued analysis of this marker.

### Subgroup analysis

**Fig 3:**
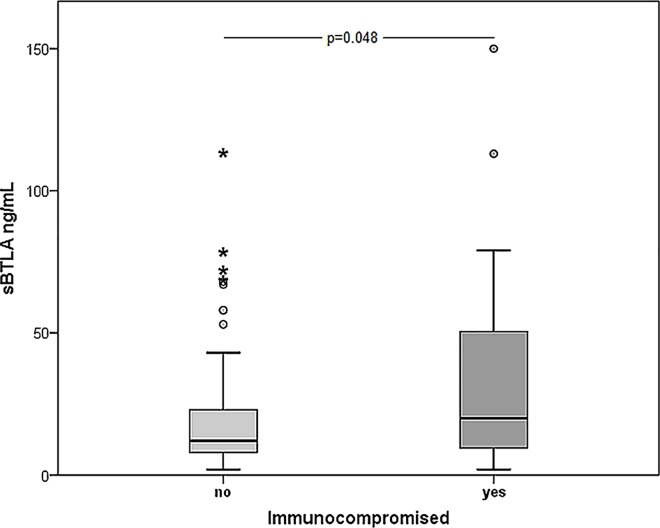
Baseline sBTLA in sepsis patients defined as immunocompromised or not at baseline. Immunocompromised (n = 27) (median 20 ng/mL, IQR, 9–55) or not (n = 71) (median 12 ng/mL, IQR 8–23) (defined by APACHE II criteria at baseline).

In the sepsis cohort no significant differences in sBTLA or sPD-1 concentrations were observed related to focus of infection, between community- or hospital-acquired infection (p = 0.6 for sBTLA, p = 0.9 for sPD-1 at baseline) or between patients with or without leukopenia (n = 18) (p = 0.5 for sBTLA, p = 0.2 for sPD-1). Sepsis patients who were defined as immunocompromised by APAHE II criteria at the time of study inclusion had significantly higher levels of sBTLA at baseline compared to those who were not (p = 0.048) (Fig 3).

There was no significant difference in sPD-1 concentrations in this respect (p = 0.4).

### Correlation between sBTLA and sPD-1 and other biomarkers

**Fig 4:**
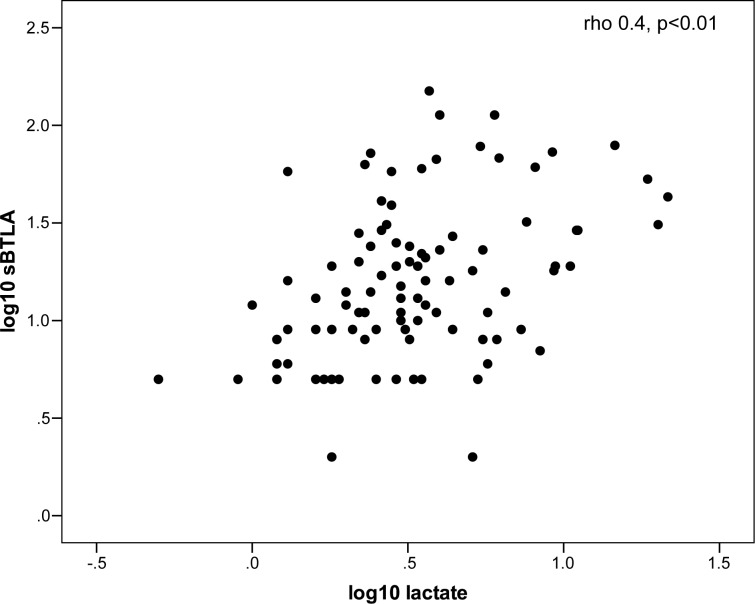
Correlation between baseline sBTLA and lactate in the sepsis cohort. Data is presented on log10 scales due to non-Gaussian distribution of both sBTLA and lactate.

sBTLA and sPD-1 were not correlated (Spearman’s rho -0.08, p = 0.06). Correlations were analyzed between baseline concentrations of the studied markers and leukocyte count, CRP and lactate levels. In the sepsis cohort sBTLA levels correlated moderately to lactate (Fig 4).

No other significant correlations between sBTLA or sPD-1 and the above-mentioned markers were found.

### Markers of severity of disease and prognosis in sepsis

**Fig 5:**
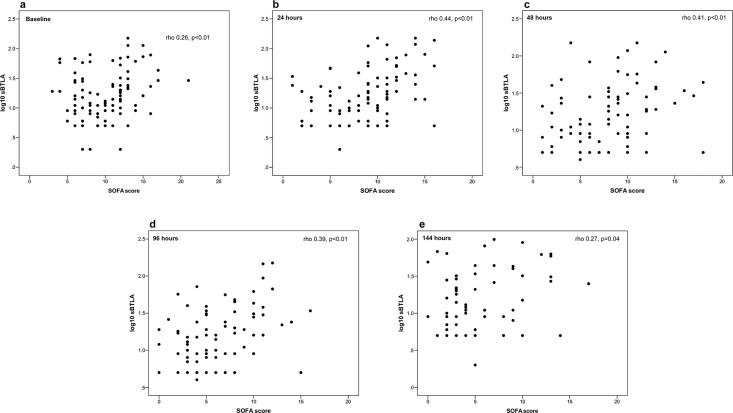
Correlation between sBTLA and SOFA score in sepsis patients. a) baseline b) 24 hours c) 48 hours d) 96 hours and e) 144 hours. sBTLA is presented on log10 scale due to non-Gaussian distribution.

sBTLA levels correlated positively to SOFA-score, moderately at 24 and 48 hours and weakly at other time points, but no correlation was found to APACHE score (rho 0.1, p = 0.3) (Fig 5A–5E).

**Fig 6:**
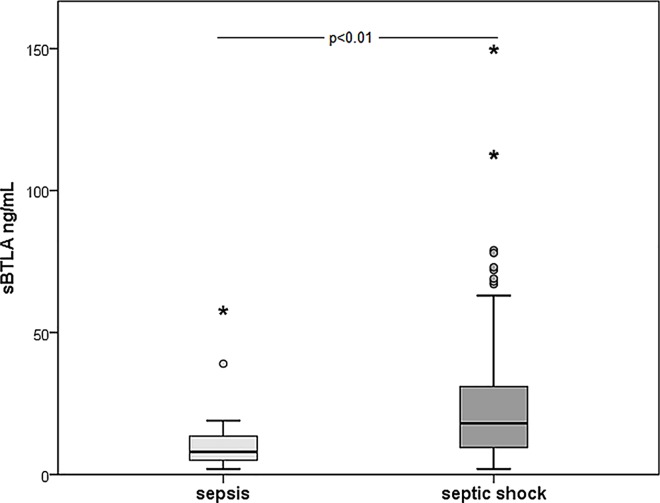
Baseline sBTLA in sepsis compared to septic shock. Patients are defined according to Sepsis-3. Sepsis (n = 23), median 8 ng/mL, IQR 5–14 vs septic shock (n = 75), median 18 ng/mL, IQR 9–31.

There was no difference in baseline sBTLA concentration between patients with severe sepsis and septic shock (SIRS-based sepsis definitions) (p = 0.68). sBTLA concentrations were however higher in patients with septic shock than in those with sepsis (Sepsis-3 definitions) (p<0.01) (Fig 6).

**Fig 7:**
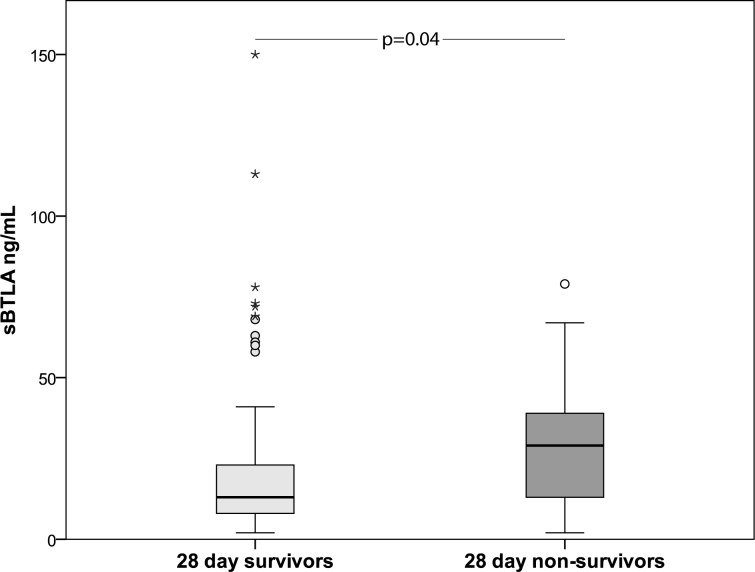
sBTLA in sepsis 28 day survivors and non-survivors. sBTLA levels at baseline in 28 day sepsis non-survivors compared to survivors: 28 (IQR 13–41, n = 17) vs 13 (IQR 8–23, n = 81) ng/mL.

18 subjects in the sepsis cohort died within 28 days after study inclusion. sBTLA levels were significantly higher at baseline in 28 day sepsis non-survivors compared to survivors (Fig 7).

**Fig 8:**
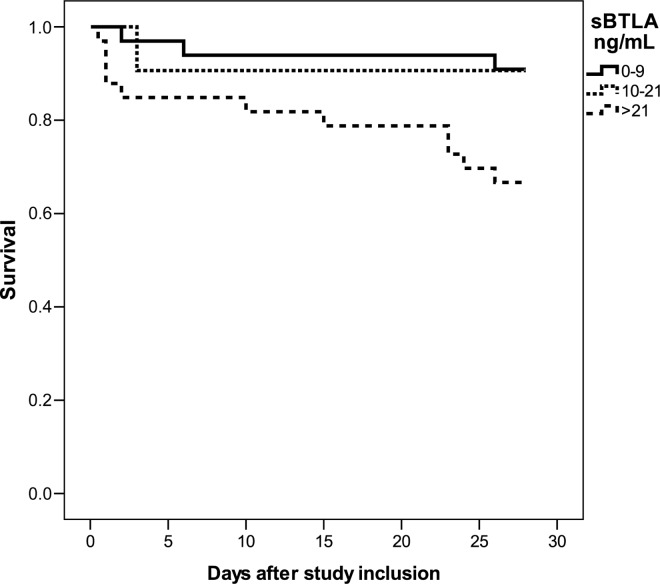
Survival curves for patients in the sepsis cohort at different baseline sBTLA concentrations. Survival curves based on the 33^rd^ and 67^th^ percentile of baseline sBTLA concentration day 0–28 after study inclusion.

Survival analyses, estimated with Cox regression with adjustment for age, showed a non-significant relative risk of death within the first 28 days of nearly 3 per log unit increase in baseline sBTLA concentration (HR 2.7 (0.9–8.4) p = 0.08), AUC 0.66 (95% CI 0.51–0.82), and a similar association after adjustment for comorbidities. There was, however, a significant 5-fold higher relative risk of death in patients with a high (> 21 ng/mL) compared to those with a low (<9 ng/mL) baseline plasma sBTLA, with adjustment for age and comorbidites (HR 5.1, 95% CI 1.3–19, p = 0.02), but no increased risk for patients with intermediate (10–21 ng/mL) compared to low baseline sBTLA (HR 1.2, 95% CI 0.2–6.2, p = 0.81) (Fig 8).

With a cut-off at 21 ng/mL, and after adjustment for age and comorbidities, a significant association was seen between the higher level of baseline sBTLA and mortality (HR 4.6, 95% CI 1.6–12.8, p<0.01, compared to <21 ng/mL). The association remained significant after adjustment for APACHE II score (HR 4.8, 95% CI 1.7–14, p<0.01). The association between baseline log10 sBTLA and mortality disappeared when adjustment was made for log10 lactate (HR 1.3 (95% CI 0.3–4.9, p = 0.7), but if categorized as high-low, HR for sBTLA >21 ng/mL was 2.9 (95% CI 0.9–8.7), p = 0.06 after adjustment for lactate. Time-varying Cox Regression showed an even stronger and significant association between sBTLA and mortality, both when evaluated on a continuous log-10 scale and as a categorical (high vs. low) marker. Adjustment for s-lactate was not possible in these models due to missing data (Table 2).

**Table 2:** Relative risk for 28 day mortality in the sepsis cohort.

		HR (95% CI)		
	Sepsis cohort (n = 98, 17 events)	adjusted for age	age + comorbidities	age + comorbidities + lactate (n = 96)
**Baseline measure analyses**				
**BTLA continuous**^**1**^	1.2 (0.4), 14 (8–29)	2.7 (0.9–8.4) p = 0.08	2.6 (0.8–8.2) p = 0.09	1.3 (0.3–4.9) p = 0.70
**BTLA low ≤9 ng/mL**	33 (9%)	ref	ref	ref
**BTLA med 10–21 ng/mL**	32 (9%)	1.2 (0.2–5.8) p = 0.85	1.2 (0.2–6.2) p = 0.81	0.8 (0.1–4.1) p = 0.80
**BTLA high >21 ng/mL**	33 (33%)	5.0 (1.3–18) p = 0.02	5.1 (1.3–19) p = 0.02	2.6 (0.6–11) p = 0.19
**BTLA low-med <21 ng/mL**	65 (9%)	ref	ref	ref
**BTLA high >21 ng/mL**	33 (33%	4.6 (1.7–13) p<0.01	4.6 (1.6–12.8) p<0.01	2.9 (0.9–8.7) p = 0.06
**s-lactate continuous**^**1**^	0.5 (0.3), 3.2 (2.2–5.2)	38 (7.0–204) p<0.01	35 (6.8–179) p<0.01	31 (5.6–175) p<0.01^3^
**Cardiovascular disease no**	88 (18%)	ref		
**Cardiovascular disease yes**	10 (10%)	0.4 (0.06–3.3) p = 0.43		
**Congestive heart failure no**	93 (16%)	ref		
**Congestive heart failure yes**	5 (40%)	2.1 (0.5–9.8) p = 0.32		
**COPD no**	94 (18%)	ref		
**COPD yes**	4 (0%)	NA		
**Renal failure no**	95 (17%)	ref		
**Renal failure yes**	3 (33%)	2.5 (0.3–19) p = 0.37		
**Diabetes no**	82 (16%)	ref		
**Diabetes yes**	16 (25%)	1.4 (0.4–4.2) p = 0.57		
**Liver disease no**	91 (19%)	ref		
**Liver disease yes**	7 (0%)	NA		
**Malignancy no**	64 (16%)	ref		
**Malignancy yes**	34 (21%)	1.3 (0.5–3.5) p = 0.56		
**0 comorbidity**	48 (15%)	ref		
**1 comorbidity**	31 (16%)	1.1 (0.3–3.4) p = 0.90		
**≥2 comorbidities**	19 (26%)	1.6 (0.5–5.0) p = 0.44		
**APACHE II, mean (SD)**	22 (8)	1.07 (1.01–1.13) p = 0.01	1.07 (1.01–1.13) p = 0.01	
**PD1 continuous**^**1**^	3.1 (0.5), 1.3 (0.6–2.8)	0.9 (0.3–2.4) p = 0.84	0.9 (0.3–2.4) p = 0.82	
**PD1-1 low <0.85 ng/mL**	33 (18%)	ref	ref	
**PD-1 med 0.85–2.0 ng/mL**	32 (16%)	0.9 (0.3–3.0) p = 0.87	0.9 (0.3–3.0) p = 0.88	
**PD-1 high >2.0 ng/mL**	33 (18%)	1.0 (0.3–3.2) p = 0.98	1.0 (0.3–3.1) p = 0.98	
**Time varying analyses**^**2**^				
**BTLA continuos (log10)**		8.7 (3.1–25) p<0.01	9.6 (3.0–31) p<0.01	
**BTLA low 0–9 ng/mL**		ref	ref	
**BTLA med 10–21 ng/mL**		0.8 (0.1–8.7) p = 0.86	0.8 (0.1–9.0) p = 0.86	
**BTLA high >21 ng/mL**		9.0 (2.1–39) p<0.01	9.2 (2.1–40) p<0.01	
**BTLA low-med ≤21 ng/mL**		ref	ref	
**BTLA high >21 ng/mL**		9.9 (2.8–35) p<0.01	10 (2.8–35) p<0.01	

Data is presented as n (% 28 day mortality) unless otherwise stated.

ref = reference.

^1^ (log 10) mean (SD), median (IQR).

^2^ 53/461 sBTLA measurements are imputed.

^3^ Adjusted for age, comorbidities and log10 sBTLA continuous scale. If adjusted for sBTLA >21 ng/mL: HR 20 (95% CI 3.7–111, p<0.01).

AUC for baseline sBTLA was 0.66 (95% CI 0.51–0.82) and for lactate 0.71 (95% CI 0.55–0.87).

AUC for sBTLA>21 ng/mL was 0.69 (95% CI 0.54–0.83).

In ICU controls 28 day mortality was 39%. There was no difference in sBTLA concentration between survivors and non-survivors among ICU controls (at baseline p = 0.5). sBTLA >21 ng/mL at baseline showed no significant age-adjusted association to mortality (HR 1.6, 95% CI 0.4–6.5, p = 0.5) and sBTLA levels did not correlate significantly to SOFA score in this cohort (at baseline rho 0.3, p = 0.1).

sPD-1 concentrations did not differ significantly between subgroups according to old (severe sepsis-septic shock) or new (sepsis-septic shock) sepsis definitions (p = 0.2 and p = 0.7 respectively) or between 28 day survivors and non-survivors in the sepsis cohort (p = 0.9) and ICU controls (p = 0.7), nor did they correlate to markers of disease severity (data not shown). There was no difference in risk of death between low versus intermediate and high levels of sPD-1 at baseline (adjusted for age) in either the sepsis (high vs low: HR 1.0, 95% CI 0.3–3.2 p = 0.98) or the ICU control cohort (data not shown).

### Age and sex

In ICU controls, but not in the sepsis cohort, sBTLA was lower in subjects over 65 years of age at baseline (5 ng/mL, IQR 2–8 vs 11, IQR 9–40, p<0.01). There was no significant correlation between baseline sBTLA and age in the sepsis cohort (rho -0.1, p = 0.3). Only one HC was above 65 years of age, hence age group comparison was not possible, but there was no significant correlation between sBTLA and age in this cohort (rho -0.08, p = 0.7). PD-1 concentrations did not correlate to age (Spearman’s rho -0.09, p = 0.3). There was no statistically significant difference related to sex between study cohorts in the concentration of the studied markers (data not shown).

## Discussion

In the present study we found that plasma concentrations of the soluble isoform of the co-inhibitory receptor B- and T-lymphocyte Attenuator (sBTLA) were low in healthy individuals and elevated early in critical illness, most markedly in sepsis. We found a correlation to severity of disease and, notably, a nearly 5-fold increase in mortality for sepsis patients with a baseline sBTLA concentration of >21 ng/mL compared to those with a concentration below that threshold. The association with mortality remained consistent after adjustment for comorbidities, but decreased after adjustment for baseline lactate concentration. The ability to diagnose sepsis in the critically ill cohort was however low. Evaluation of sBTLA as a dynamic marker confirmed an association to mortality. Unlike sBTLA, Programmed Death 1 (sPD-1) concentrations were lower in patients with sepsis and septic shock than in healthy control subjects, with no correlation to disease severity, and neither sPD-1 was a good biomarker to diagnose sepsis in critically ill patients. Soluble Cytotoxic T Lymphocyte-associated Antigen 4 (sCTLA-4) concentrations were low or undetectable.

This is to the best of our knowledge the first study of soluble co-inhibitory molecules in sepsis. It is not known if or how the plasma concentrations correlate to the expression of the membrane-bound correspondent receptors. Previous studies on sPD-1 have shown elevated serum and plasma levels and a correlation to disease activity in for example rheumatoid arthritis and systemic sclerosis, which is interesting in light of the observation that PD-1 deletion results in susceptibility to autoimmune disease in mice, but on the other hand sPD-1 was decreased in idiopathic thrombocytopenic purpura. sPD-1 in plasma has also been reported to be associated with development of hepatocellular carcinoma in chronic hepatitis B [15, 34–37]. Considering that PD-1 is not expressed on naïve T cells, and that there are some earlier reports of up-regulated peripheral blood T cell expression of PD-1 in septic shock, it was surprising to find plasma sPD-1 to be lower in patients with sepsis than in HC. In the early phase of sepsis there is a decrease in the absolute number of circulating lymphocytes as demonstrated for example by Boomer et al [20]. It is possible that this could explain why soluble PD-1 is decreased while PD-1 expression is increased on circulating T cells. Unfortunately we had incomplete data on lymphocyte count and could therefore not evaluate if sPD-1 levels correlated to lymphocyte levels. It has been demonstrated that sPD-1 can block the inhibitory effect of membrane-bound PD-1 on T-cell activation in vitro [38]. The observed decrease of plasma soluble PD-1 in the sepsis cohort could be explained by sPD-1 consumption upon immune activation as a result of ligation to PD-L1 and–L2, resulting in a loss of T cell inhibition in the acute phase of infection. It is also possible that a pro-inflammatory stimulus causes a switch in PD-1 gene transcription in favor of the membrane-bound isoform. Interfering with PD-1-PD-L1/PD-L2 interaction has been proposed as a method of counteracting immunosuppression in sepsis, and animal models have shown positive results in terms of immune cell function and survival [22, 23]. We found no evidence for an upregulation of sCTLA-4 in sepsis. Previous studies on soluble CTLA-4 show increased plasma concentrations in several autoimmune disorders, but low or undetectable levels in healthy individuals [39–42]. There might be certain pathogenic mechanisms present in autoimmune disease, but not in sepsis, resulting in an increased production of sCTLA-4. Perhaps due to the limited sensitivity of commercially available ELISA kits, sCTLA-4 is difficult to study.

Unlike for sPD-1 and sCTLA-4 there are no publications on sBTLA in other disease states. The existence of sBTLA in sera from healthy individuals has been demonstrated by Wang et al [43]. In their study, but not in ours, sBTLA was positively correlated with age. Studies on cell expression of BTLA in sepsis show conflicting results. Shubin and co-workers found the percentage of peripheral blood CD4^+^ T cells expressing BTLA to be higher in patients with sepsis than in patients with non-infectious SIRS, while Boomer et al recorded no difference between patients sampled within 24 hours after sepsis onset and healthy controls (HC) [20, 21]. In the Shubin study, patients were however sampled later in the course of sepsis which makes comparison difficult. In the latter study, T cell expression of BTLA was only 50% in HC while Boomer et al found it to be >90% in both septic patients and HC. A recent study by Shao et al, where patients were sampled within 24 hours after Emergency Department admission, showed 90% BTLA+ CD4^+^ T cells in healthy control subjects, with a lower expression in patients with sepsis, even lower in severe sepsis and lowest in septic shock, [44]. Besides higher expression of BTLA in sepsis, Shubin and co-workers reported a higher proportion of CD4+ T cells expressing BTLA in patients who subsequently developed a nosocomial infection [21]. Although many of the ICU control subjects in our study developed an infection during their ICU stay, we did not analyze this further due to the small number of subjects in this cohort.

The BTLA-HVEM pathway is complicated by the fact that HVEM has multiple partner molecules through which cross-linking results in either a co-stimulatory (LIGHT and LTα) or a co-inhibitory (BTLA and CD160) signal [14]. Therefore BTLA might be a more fine-tuned immune regulator than CTLA-4 and PD-1. The origin of sBTLA remains to be elucidated. Perhaps it is generated through alternative splicing of mRNA as has been demonstrated for sPD-1 and sCTLA-4 [26, 27, 45]. Another potential source of the measured BTLA is proteolytic cleavage of the outer part of the membrane-bound receptor, possibly representing a means of controlling T cell inhibition. Thirdly, although specific antibodies to detect sBTLA and sPD-1 have been developed, at the time of carrying out these experiments there were no commercially available ELISA kits able to discriminate soluble BTLA and PD-1 from the membrane bound isoform, and we cannot exclude that some BTLA and PD-1 detected in the analyses may be connected to cell fragments in plasma [46]. In this study, baseline plasma sBTLA concentrations were highest in the sepsis cohort suggesting that sBTLA is a marker for an activated pathway in the immune system, triggered by infection. sBTLA concentrations and disease severity in the sepsis cohort were associated; sBTLA and SOFA score were correlated and septic shock patients had higher concentrations than those with sepsis without shock. Moreover, we found an association between sBTLA and 28 day mortality for patients in the sepsis cohort, when assessed by baseline value, and, with reservation for the possible bias of the imputation method used for missing values, on an even stronger level when evaluated as a dynamic marker, indicating that repeated measurements of sBTLA during the course of sepsis may be valuable. In keeping with our findings, it was shown in an experimental sepsis model that BTLA-deficient mice were protected from kidney injury and had a higher rate of survival [47]. However, in a recently published study by the same group, anti-BTLA antibody administered intraperitoneally directly after cecal ligation and puncture in mice showed opposite effects [48]. Taken together, our results suggest that, although soluble co-inhibitory receptors do not appear to be good markers to diagnose sepsis in the critically ill patient, sBTLA may be of interest as a prognostic indicator, perhaps in combination with lactate and other biomarkers, and that further studies on the immune pathways involving sBTLA may be of value in clarifying the pathogenesis in human sepsis.

One factor of uncertainty, in this and other sepsis studies, is that the course of sepsis differs between patients and infecting agents. The time point of onset of infection can also be difficult to determine, which was the case in 12/101 sepsis patients in this study. In patients where it could be defined, the delay from the first symptoms of an infection and drawing of the first blood sample was in median 2 days (IQR 1–3, range 0–19). This study has other limitations. There is a substantial shortfall in plasma samples in the latter half of the study period, due to missing samples or patients being transferred from the ICU because of recovery or for other reasons, resulting in changed composition of the study group as well as a significantly reduced number of ICU controls, adding some difficulty to drawing conclusions on the dynamics of the studied markers. Furthermore almost half of the ICU controls developed an infection during the study period. To reduce bias due to these factors, we limited group comparison of the sepsis and ICU control cohorts and sepsis survivors and non-survivors to the baseline measurement. Considering the relatively few study subjects and the fact that there are no previous publications on this marker in sepsis patients, the chosen threshold sBTLA concentration for increased risk of mortality may not be optimal and alternative threshold values should be tested in future studies.

Finally, this is an observational study and no conclusions can be drawn in terms of the underlying immunological mechanisms. Future mechanistic studies are required to examine the biological activity of soluble BTLA and why high levels are associated with mortality. This may also clarify whether receptor intervention could be favorable in human sepsis or if sBTLA will serve better as a biomarker.

Our results suggest that sBTLA may be valuable as a dynamic prognostic marker in sepsis. However, additional studies, including patients with less severe disease, and ideally with correlation to known markers of sepsis-induced immunosuppression, are needed to evaluate the biological significance of sBTLA and whether it may be useful, possibly in conjunction with other biomarkers for risk stratification in the septic patient.

## Supporting Information

S1 File Data. Data file containing all raw data used in the statistical analyses.
(XLSX)
